# National survey of smoking cessation provision in China

**DOI:** 10.18332/tid/104726

**Published:** 2019-04-02

**Authors:** Haoxiang Lin, Dan Xiao, Zhao Liu, Qiang Shi, Peter Hajek, Chen Wang

**Affiliations:** 1 Center for Respiratory Diseases, China-Japan Friendship Hospital, Beijing, China; 2 WHO Collaborating Centre for Tobacco Cessation and Respiratory Diseases Prevention, China-Japan Friendship Hospital, Beijing, China; 3 Tobacco Medicine and Tobacco Cessation Center, China-Japan Friendship Hospital, Beijing, China; 4 National Clinical Research Center for Respiratory Diseases, Beijing, China; 5 Wolfson Institute of Preventive Medicine, Queen Mary University of London, London, United Kingdom; 6 Chinese Academy of Medical Sciences & Peking Union Medical College, Beijing, China

**Keywords:** smoking cessation, China, cessation clinic

## Abstract

**INTRODUCTION:**

Treatment for smoking cessation is an important part of tobacco control and has been promoted within the Chinese health service for many years. The aim of this study was to assess the current status of smoking cessation treatment provision within the Chinese health service.

**METHODS:**

A nationwide survey, sponsored by the National Health and Family Planning Commission, assessed smoking cessation activities in all 31 Provincial Health and Family Planning Commissions (PHFPCs) in China. Within the 31 provinces, 366 hospitals and primary care centers running smoking cessation clinics provided details of their activities.

**RESULTS:**

Findings show that all PHFPCs took steps to promote smoking cessation, such as by conducting inspections and supervising local cessation clinics. Specifically, among the 366 health institutions,73% were based in general hospitals, with smoking cessation clinics predominantly located in respiratory departments. Furthermore, only 43% provided smoking cessation medications.

**CONCLUSIONS:**

This was the first nationwide survey of smoking cessation support available to smokers in China. It provides the most comprehensive picture of the treatment arm of smoking cessation activities so far. The Chinese government has taken measures to support smoking cessation, however, further efforts are needed to address the imbalanced distribution of resources and the limited availability of medications. On-going monitoring of barriers and facilitators affecting treatment provision is needed, as well as an understanding of the importance of each hospital focusing on working priorities specific to their needs. This survey could be a reference for other countries starting to promote smoking cessation.

## INTRODUCTION

In China, cancer has been the leading cause of death since 2010 and a major public health concern^1^. In 2015, 4.29 million new cancer cases and 2.81 million cancer deaths were recorded, with lung cancer being the most common^2^. Of these deaths, 23% to 25% were attributable to smoking^2^. Smoking cessation treatment has been shown to be effective in cancer prevention^3^ and cancer patient survival^4^. However, if smoking cessation efforts do not become widespread, smoking related deaths will continue to rise; estimated from 1 million in 2010 to 2 million by 2030^5^.

In 2015, the China Adult Tobacco Survey found that there were 316 million smokers (52.1% of adult males and 2.7% of adult females), of which 18% were planning to quit smoking within 12 months^6^. As most self-help quit attempts fail^7^, this suggests that there are tens of millions of smokers in China who could benefit from stop-smoking treatment.

The Chinese government ratified the WHO Framework Convention on Tobacco Control (WHO FCTC) in 2005, coming into force in China in January 2006^8^. A key component of this program was offering help to smokers (Article 14)^9^, but, at that time most medical and health institutions lacked the capacity to offer smoking cessation services.

Nevertheless, The Chinese Government’s Tobacco Control Plan (2012–2015) recognized this problem and adopted measures to improve treatment access. Chinese Smoking Cessation Guidelines were issued in 2007 and 2015^10^, and provincial governments received funding to establish smoking cessation clinics in 2014. From 2006, smoking cessation training was incorporated into the medical education system as a chapter in the book of *Respiratory Medicine* and *Internal Medicine*, as well as tobacco control content added into the national qualification examination for medical practitioners.

Previous studies have only covered a few areas or collected insufficient information^11,12^. As a result, to monitor progress The National Health and Family Planning Commission (NHFPC, formerly Ministry of Health) authorised a nationwide survey to assess the state of smoking cessation provision across China from 2016 to 2017.

This study reports the first nationwide survey of smoking cessation support available in China. It provides baseline data and a comprehensive picture of the treatment arm of tobacco control activities available so far. Importantly, it identifies issues and remedies not only useful to China but also to other countries that are embarking on providing smokers with treatment options.

## METHODS

### Study design

This was a national survey covering all 31 provinces of mainland China. It was conducted by WHO Collaborating Center for Tobacco Cessation and Respiratory Diseases Prevention at the China–Japan Friendship Hospital in Beijing. The questionnaire and online platform were developed in July–September 2016, and the survey was carried out between October 2016 and March 2017.

### Measures

**Figure 1:**
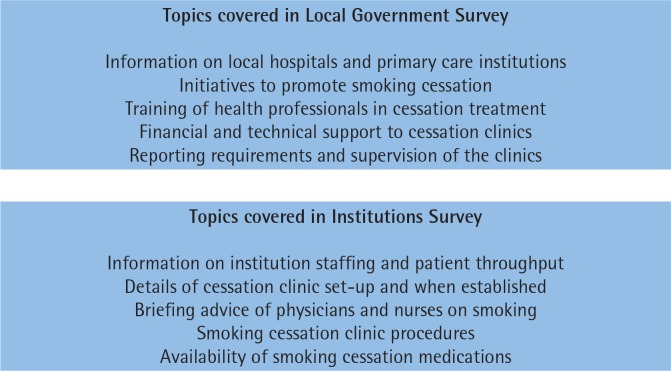
Questionnaire contents

Two questionnaires were developed using previous survey frameworks, proven to be effective internationally^13,14^. One assessed activities at local government level (Government Survey, GS) and the other assessed smoking cessation provision at individual institutions (Institution Survey, IS). The questionnaires comprised mostly multiple-choice questions. The GS consisted of 18 questions. These included support for smoking cessation at government level, policy development, capacity building, and technical and financial support from Provincial Health and Family Planning Commissions (PHFPCs) and relevant provincial governments. The IS consisted of 26 questions concerning settings of the clinics, staff training, medical resources, and treatment details. Additional reference materials were used to develop questions regarding treatment details and medical resources. These included a tobacco dependence survey conducted in 121 countries by The University of Nottingham^13^, and a related paper published by West et al.^14^. Further questions were created in collaboration with the NHFPC of China by taking into consideration China’s specific status. The contents of the questionnaires are outlined in Figure 1.

Institutions responding to IS were divided into two groups: those that included staff that had undergone specialist training in smoking cessation offered nationally by the WHO Collaborating Center at China–Japan Friendship Hospital in Beijing (Participating Institutions, PI) and those that did not (Non-participating Institutions, NPI). The training comprised a 3-day course using the model of Withdrawal Oriented Treatment practiced by the UK Specialist Stop Smoking Service^15^. This was adapted to accommodate the healthcare environment in China and on-going support from the Beijing team^16^.

### Procedures

NHFPC issued a formal request to all Chinese PHFPCs to complete the Local Government Survey (GS), and appoint a coordinator to liaise with the research team to ensure all eligible local healthcare institutions complete the Institution Survey (IS). The GS questionnaire was completed by the 31 PHFPCs responsible for tobacco control. The IS questionnaire was completed by smoking cessation doctors or counselors in hospitals in mainland China.

To ensure participants could access the survey, the questionnaires were placed online and accessible via the link http://112.124.38.62/jymz/. The appropriate questionnaire was displayed automatically based on the category of the user’s login. In the meantime, all focal persons in the eligible local healthcare institutions were informed via telephone. Following this, both the coordinator and research team in Beijing monitored progress by the online system. Monitoring included: how many hospitals had registered, how many registered institutions had submitted questionnaires, and how many had been approved by the research team.

### Quality assurance

The self-check function of the online survey system automatically identified missing data, logical errors and illegal characters. Completed questionnaires were further reviewed by the study staff who then contacted respondents for clarifications if any problems were detected. Local coordinators reviewed the list of respondents to ensure all healthcare institutions that operated smoking cessation clinics within their jurisdiction were included.

### Statistical analysis

Descriptive statistics on continuous variables are reported as means and standard deviations. Categorical variables are presented as counts and percentages. Comparisons between institutions that included trained staff were performed and the rest used chi-squared tests with odds ratios and 95% confidence intervals. No imputation was performed for missing data.

## RESULTS

### Local government survey

The response rate was 100%, with all 31 PHFPCs in China responding to the survey.

All PHFPCs took steps to promote smoking cessation using mass media to encourage smokers to quit, such as smoking cessation related PSA, micro film and TV shows. Of the PHFPCs, 77% published locally relevant government recommendations or documents targeting health institutions to promote cessation treatment and patient referrals. Additionally, 77% commissioned local cities to promote smoking cessation, and 97% funded or supported cessation publicity on World No-Tobacco Day. In total, 84% of PHFPCs organized smoking cessation training for physicians within the past three years.

One of the tasks of PHFPCs is to conduct inspections and supervision of local health facilities. At present, most include local cessation clinics. Specifically, 55% cover smoking cessation facilities in their jurisdictions, 42% cover only a sample and 3% do not include inspection and supervision in their activities within the past three years. Moreover, 52% requested no reports from the local smoking cessation clinics, 23% monitored the clinics annually, and 26% requested quarterly or monthly reports.

All PHFPCs had smoking cessation clinics in their localities, but only 9 (29%) provided direct financial support for the clinics within the past five years. Furthermore, in two provinces the clinics received municipal support, however, in the remaining 20 there was no dedicated financial support provided for smoking cessation treatment.

The responsibility for tobacco control within PHFPCs resides with the deputy directors, of whom 23% were current smokers and 10% of director generals were current smokers.

### Institution survey

The survey identified 366 institutions that provided smoking cessation treatment via dedicated clinics in mainland China. The Supplementary Table shows their distribution across the 31 provinces of China.

**Figure 2:**
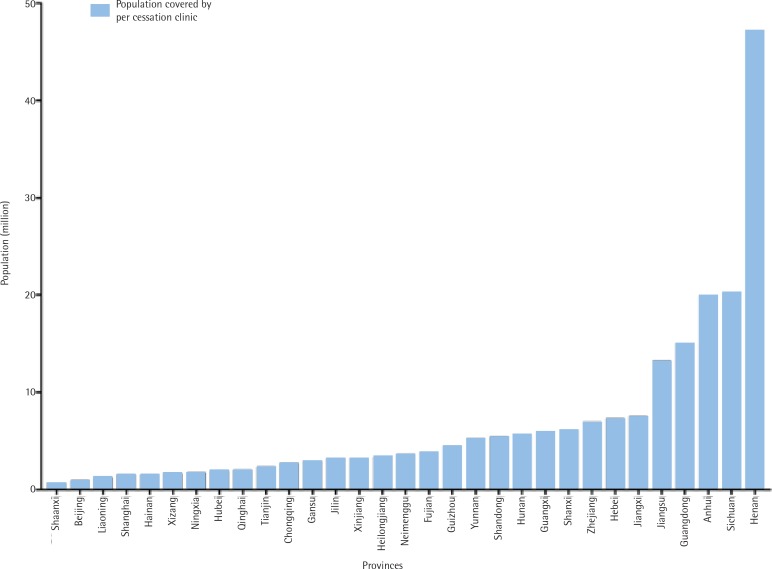
Population covered by each cessation clinic

The population of each clinic, per province covered, was estimated using the 2010 population data of the National Bureau of Statistics of China^17^. Figure 2 shows that five of the 31 regions have much more limited coverage than the rest of the country.

### Characteristics of institutions that host smoking cessation clinics

A total of 266 clinics were located in general hospitals, 36 in specialised hospitals, 33 in traditional Chinese medicine hospitals, and 9 in hospitals that provided both traditional Chinese and Western medicine. Moreover, 1 was located in a Tibetan medicine hospital, 11 in community health centers, and 10 in township health centers. Most institutions were tertiary or secondary hospitals (Table 1).

**Table 1:** Characteristics of institutions that established smoking cessation clinics

*Questions*	*Number (%) (N=366 )*
**Type of health institutions**	
General hospital	266 (72.7)
Specialised hospital	36 (9.8)
Traditional Chinese medicine hospital	33 (9.0)
Community health service center	11 (3.0)
Township health service center	10 (2.7)
Traditional Chinese and Western medicine hospital	9 (2.5)
Tibetan medicine hospital	1 (0.3)
**Level of health institutions**	
Tertiary hospital	217 (59.3)
Secondary hospital	122 (33.3)
Primary care institution	13 (3.6)
Primary hospital	9 (2.5)
Others	5 (1.4)
**Affiliation of cessation clinics**	
Department of respiratory medicine	230 (62.8)
Department of general medicine	29 (7.9)
Department of psychiatry or psychological consultation	17 (4.6)
Department of cardiology or cardiovascular surgery	9 (2.5)
Department of traditional Chinese medicine	9 (2.5)
Independent department	3 (0.8)
Other departments	69 (18.9)

Clinics were most frequently located in respiratory departments, general clinics and psychology or psychiatry departments.

**Figure 3:**
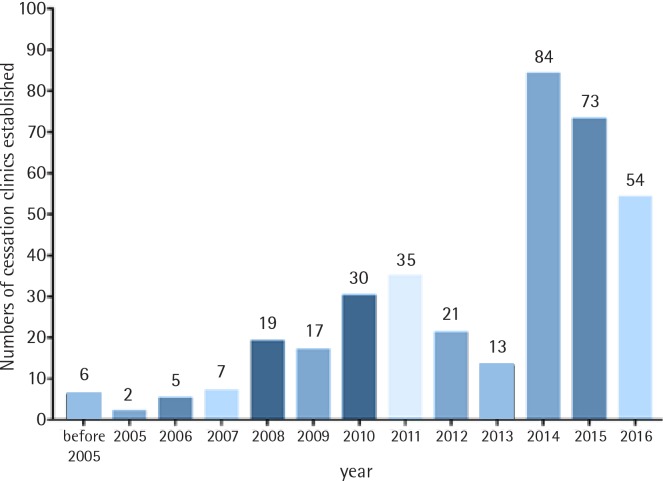
Date when smoking cessation clinics were established

Figure 3 shows the dates when the clinics were established and provides a succinct history of smoking cessation provision in China. After the national program was launched in 2006, the number of clinics kept increasing. The dramatic increase in 2014 occurred when the National Government started to provide financial support to smoking cessation clinics.

### Treatment provided

Regarding treatment, 351 (96%) of the clinics provided individual counseling (with 5% also offering group treatment). However, only 157 (43%) provided smoking cessation medications (Varenicline, Bupropion or nicotine replacement treatment (NRT), with the nicotine patch most commonly used for NRT in China). In addition, 9 (2.5%) of the clinics provided acupuncture.

**Figure 4:**
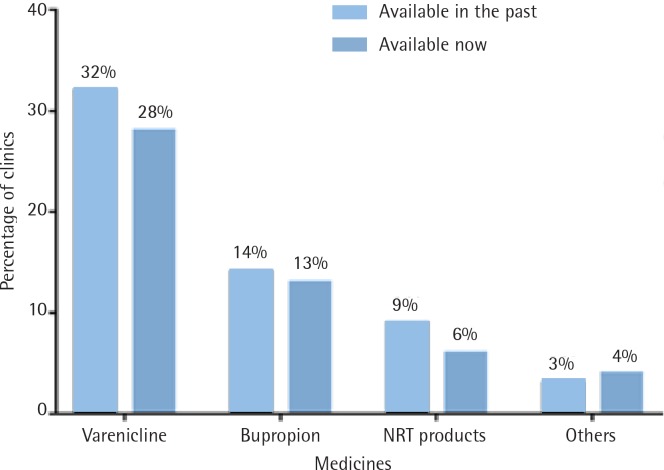
Smoking cessation medications provided at present and in the past (% of 366 clinics)

Figure 4 shows the provision of smoking cessation medications in 2016–2017 compared with medication provided in previous years. As shown, Varenicline is the most widely provided medication, however, the overall use of medication is low and in further decline

(p<0.05 for change in Varenicline use).

Data on the number of patients who attend the clinics and treatment outcomes are currently not routinely collected by all the clinics.

### Differences between tertiary hospitals and others

Comparison of the different level hospitals showed that tertiary hospitals were more likely to record patients’ comprehensive information at their first visit (p<0.01), and provide additional treatment options (p<0.01). Although the availability of smoking cessation medication is low, more tertiary hospitals have Varenicline (p<0.01) and CO monitors (p<0.01). However, specialist cessation training (p<0.01) and follow-up treatment was performed poorly in hospitals at all level (p<0.05) (Table 2).

**Table 2:** Treatment information between tertiary hospitals and others^a^

	*Tertiary hospitals n (%)*	*Others n (%)*	*Difference n (%)*	*Total n (%)*
Have Varenicline	96 (44.2)	20 (13.4)	30.8**	116 (31.7)
Have Bupropion	36 (16.6)	16 (10.7)	5.9	52 (14.2)
Have NRT products	20 (9.2)	11 (7.4)	1.8	31 (8.5)
Have at least one cessation doctor with specialist treatment training	38 (17.5)	11 (7.4)	10.1**	49 (13.4)
Record patients’ comprehensive information at their first visit	129 (59.4)	43 (28.9)	30.5**	172 (47.0)
Can provide individual counseling	210 (96.8)	141 (94.6)	2.2	351 (96.0)
Can provide behavior support	176 (81.1)	100 (67.1)	14.0**	276 (75.4)
Can provide smoking cessation medications	120 (55.3)	37 (24.8)	30.5**	157 (42.9)
Arrange 4–6 times follow-up within six months	31 (14.3)	11 (7.4)	6.9*	42 (11.5)
Have CO monitor	**114 (52.5)**	**30 (20.1)**	32.4**	144 (39.3)

a Others include secondary and primary hospitals, and primary care institutions.

** p<0.01

* p<0.05

### Differences between PI and NPI institutions

Chinese Smoking Cessation Guidelines (2015) provide a number of recommendations. These include, recording patients’ smoking status at their first visit, delivering brief smoking cessation advice at all hospital clinics, providing stop-smoking medications, arranging a series of follow-up visits and monitoring smoking status using carbon monoxide (CO) monitors at specialist clinics^10^.

**Figure 5:**
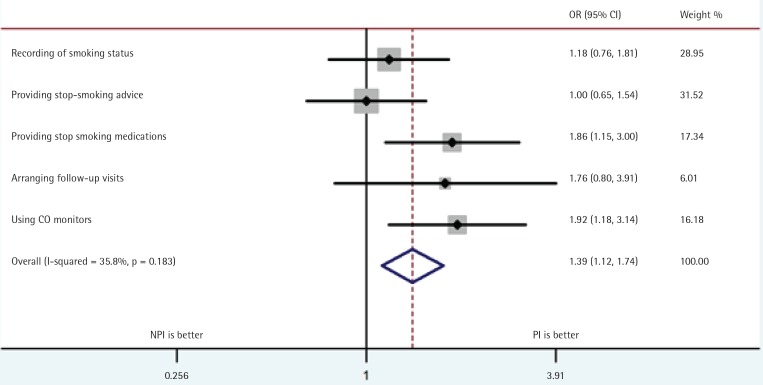
Adherence to guidelines recommendations in PI and NPI groups

Comparison of PI and NPI institutions showed that the PI were more likely to provide medication to smokers and use CO monitoring, as well as record patient smoking status and arrange regular follow-up visits (Figure 5).

## DISCUSSION

This was the first nationwide survey of smoking cessation support available to smokers in China. With 100% response rate from local government bodies, it provides the most comprehensive picture of the treatment arm of smoking cessation activities available so far.

The survey does have several limitations. First, it was not possible to objectively verify the survey answers. This is unlikely to pose a major problem, but the representatives of some institutions responding may not have full details of their smoking cessation clinics. Second, surveys can be biased due to respondents’ tendencies to provide socially desirable answers. Although, on the basis of the overall picture that the survey generated and the personal knowledge of some clinics among the survey staff, it is believed the information obtained was accurate. Third, while all local governments were covered, it may be possible that not all local smoking cessation clinics were included. With a survey of this scale, institutions may have been missed by local coordinators or elected not to join.

Despite these limitations, the survey provides new and potentially important information. It successfully shows that local governments across China have taken on board the key smoking cessation activities. These include, supporting smoking cessation publicities, encouraging smokers to quit and training doctors in providing stop-smoking advice and support. However, only a minority of local commissions provided dedicated funding for smoking cessation clinics, and although all local healthcare systems have such clinics, the coverage is insufficient. Additionally, it is concerning to find 57% of the clinics cannot provide stop-smoking medications, instead relying solely on advice and behavioral support.

The number of hospitals that have established cessation clinics has increased in China due to support from the central government, however, the distribution is imbalanced. It is worth noting, cessation clinics were mostly affiliated with tertiary or secondary hospitals, with smoking cessation treatment not yet integrated into primary care. According to WHO, brief tobacco interventions should be available in countries with health systems of all levels. However, focus on establishment in primary care settings should be a priority due to population-level interventions relying heavily on these settings^18^. In China, primary care institutions have the potential to reach most smokers. This is due to doctors, who work for these institutions, having the flexibility to conduct relatively long follow-up treatments. Therefore, it is suggested that the NHFPC should implement a health policy to integrate smoking cessation treatment into primary care systems.

Although the overall quality of cessation clinics indicates room for improvement, results show these priorities may vary depending on different institutes. For tertiary hospitals, most have enough treatment options, therefore, improving professional training and adherence to treatment guidelines (including standardised patient information record and regular follow-up visit) is the way forward. For secondary hospitals and other lower level health institutions, the limited availability of medication and lack of basic inspection equipment should be given more attention. This means that governments could predominantly provide technical support for tertiary hospitals, while supporting more aspects for secondary hospitals and lower level health institutions.

The comparison of institutions, with and without staff who have undergone specialist training (PI and NPI institutions), shows that PI provide better care than NPI. However, this result is not based on a randomised study and it is possible institutions that arranged for their staff to receive training are more organised or motivated to provide quality treatment. Nonetheless, the observed differences concern primarily activities based on skills that are the main focus of the training and it seems likely that the training was beneficial. The national training center is now planning to target regions and institutions where training is lacking.

The limited use of smoking cessation medications identified in the survey is a problem with no easy solution. The main barrier to accessing these in China is their cost. Smokers attending the clinics incur a registration fee that is largely reimbursable, but requires them to pay in full for medications, as they are not on the essential-drugs list. For Chinese smokers, the cost of these medications (some $70 to $250 for a full course) is high. An obvious remedy would be to add them to the list, but this is not currently under consideration. One possible solution would be to provide less expensive evidence-based pharmacological aids, such as cytisine^19,20^ and nortriptyline^21,22^.

The new funding for smoking cessation clinics from the central government, allocated in 2014, triggered a marked increase in smoking cessation provision. However, the initial spur of newly established clinics has slowed down. Some local governments are still not funding any such work, and over half of existing clinics have to rely on other funding sources. These findings could enable the central government to request local commissions that do not yet implement the provision to do so in future.

A further impressive finding shows that although a number of local governments monitor and supervise the work of local clinics, a standard monitoring system is lacking. Data on the number of patients who attend the clinics and on treatment outcomes are currently not routinely collected by all. Most cessation clinics in China are not independent, but affiliated to others. This means if the clinics only register basic patient information they cannot identify the accurate number of smoking cessation patients. Reliable data on patient throughput and treatment outcomes are essential to guide the service development. Therefore, a system is needed with a standard data set required from all local providers that is collated centrally. Importantly, the government should also insist health institutions collect such information routinely.

World Health Organization 2017 data showed that 169 countries (87%) provide smoking cessation services, with more than 75% of them covering costs partially or fully^23^. China has national quitlines, but all the smoking cessation medications are at the patients’ own expense. Almost all high-income countries offer some or full cost coverage of smoking cessation services. In the UK for example, smoking cessation treatment is universally available to all smokers and mainly free of charge. NRT and other smoking cessation medications are free to about half the population according to income condition^24^. More and more middle-income countries provide comprehensive cessation services, e.g. India introduced cost-covered cessation services and a toll-free quitline from 2016, both NRT and some cessation services are cost covered^23^. The proportion of the world’s population assisted by comprehensive cessation services will increase from 33% to over 50%, if China can introduce population level cost-covered cessation services^23^.

These findings have practical implications. The survey report will be disseminated to local governments and health institutes. This will aim to encourage those struggling to catch up and provide accessible treatment options and implement other key smoking cessation measures. Moreover, the WHO Collaborating Centre for Tobacco Cessation and Respiratory Diseases Prevention holds annual international training workshops, providing a good opportunity to showcase these findings to other countries. Given that there were 316 million smokers in China consuming more cigarettes than the next top 29 cigarette-consuming countries combined^25,26^, these findings may have a positive influence to reduce smoking prevalence in China and worldwide.

## CONCLUSIONS

The survey provided the first comprehensive picture of smoking cessation activities in China. It identified signs of success and on-going progress but also areas with room for improvement. Several of the problems identified are amenable to practical remedies that can now be pursued. This survey could be useful to other countries that are starting to promote smoking cessation.
