# Supplementary material for: National survey of smoking cessation provision in China
Source: Tob Induc Dis. 2019 Apr 2;17:25. doi: 10.18332/tid/104726 (PMC6751981; doi:10.18332/tid/104726)
Supplement: Supplementary file 1 [file TID-17-25-s1.pdf]

Supplement Table. Smoking cessation clinics distribution in China

Province	Number of cessation clinics	Population	Population covered by each clinic (million)
Shaanxi	60	37327378	0.6
Beijing	25	19612368	0.8
Liaoning	40	43746323	1.1
Shanghai	16	23019148	1.4
Hainan	6	8671518	1.4
Xizang	2	3002166	1.5
Ningxia	4	6301350	1.6
Hubei	32	57237740	1.8
Qinghai	3	5626722	1.9
Tianjin	6	12938224	2.2
Chongqing	11	28846170	2.6
Gansu	9	25575254	2.8
Jilin	9	27462297	3.1
Xinjiang	7	21813334	3.1
Heilongjiang	12	38312224	3.2
Neimenggu	7	24706321	3.5
Fujian	10	36894216	3.7
Guizhou	8	34746468	4.3
Yunnan	9	45966239	5.1
Shandong	18	95793065	5.3
Hunan	12	65683722	5.5
Guangxi	8	46026629	5.8
Shanxi	6	35712111	6.0
Zhejiang	8	54426891	6.8
Hebei	10	71854202	7.2
Jiangxi	6	44567475	7.4
Jiangsu	6	78659903	13.1
Guangdong	7	104303132	14.9

Anhui	3	59500510	19.8
Sichuan	4	80418200	20.1
Henan	2	94023567	47.0

These are 2010 population data downloaded from the website of National Bureau of Statistics of China: http://www.stats.gov.cn/tjsj/tjgb/rkpcgb/qgrkpcgb/201104/t20110429_30328.html

Not including Hong Kong, Macao and Taiwan.

© 2019 Lin H.
