# Supplementary material for: National survey of smoking cessation provision in China
Source: Tob Induc Dis. 2019 Apr 2;17:25. doi: 10.18332/tid/104726 (PMC6751981; doi:10.18332/tid/104726)
Supplement: Supplementary file 2 [file TID-17-25-s2.pdf]

The Provincial Coordinators

1. Hongqi ZHAO (Shaanxi, HEC)
2. Wei LIU (Liaoning, PHFPC)
3. Lina MA (Hubei, CDC)
4. Yuan CAO (Beijing, CDC)
5. Renyou CHEN (Shandong, CDC)
6. De CHEN (Shanghai, HEC)
7. Ying SU (Heilongjiang, CDC)
8. Xingyong JIANG (Hunan, PHEC)
9. Miao HE (Chongqing, PHFPC)
10. Junyi WANG (Hebei, CDC)
11. Jianqiang BIAN (Fujian, HEC)
12. Xinping LUO (Yunnan, HEC)
13. Dong LIU (Gansu, PHFPC)
14. Ye ZHANG (Jilin, PHFPC)
15. Yan WEI (Guizhou, CDC)
16. Qimeng XIONG (Guangxi, PHFPC)
17. Xiaolin WANG (Zhejiang, PHFPC)
18. Hui LI (Neimenggu, PHEC)
19. Jie ZHAO (Guangdong, PHFPC)
20. Wen MA (Xinjiang, CDC)
21. Qingyong ZENG (Jiangxi, HEC)
22. Chen QU (Jiangsu, CDC)
23. Gang WANG (Tianjin, HEC)
24. Xin WANG (Shanxi, HEC)
25. Hongmei BAI (Hainan, HEC)
26. Zhizhong LIU (Sichuan, PHFPC)
27. Baofu HE (Ningxia, PHFPC)
28. Tao HONG (Anhui, PHFPC)
29. Zhanyu MA (Qinghai, PHFPC)
30. Weifeng WANG (Henan, CDC)
31. Zhuoma LABA (Xizang, CDC)

HEC: Provincial Health Education Center. **PHFC**: Provincial Health and Family Planning Commission. **CDC**: Provincial Disease Control and Prevention Center

© 2019 Lin H.
